# Influence of brick laying height on biomechanical load in masons: Cross-sectional field study with technical measurements

**DOI:** 10.3233/WOR-230325

**Published:** 2024-09-11

**Authors:** Mikkel Brandt, Rúni Bláfoss, Markus Due Jakobsen, Afshin Samani, Jeppe Z.N. Ajslev, Pascal Madeleine, Lars L. Andersen

**Affiliations:** a National Research Centre for the Working Environment, Copenhagen, Denmark; b Research Unit for Muscle Physiology and Biomechanics, Department of Sports Science and Clinical Biomechanics, University of Southern Denmark, Odense, Denmark; c ExerciseTech, Department of Health Science and Technology, Faculty of Medicine, Aalborg University, Denmark

**Keywords:** Bricklayers, electromyography, actigraphy, musculoskeletal diseases, ergonomics, building and construction

## Abstract

**BACKGROUND:**

Work-related musculoskeletal disorders (WMSDs) located in the low back and neck/shoulder regions are major concerns for both workers, workplaces, and society. Masons are prone to WMSD, because their work is characterized by repetitive work and high physical workload. However, the knowledge on the physical workload during bricklaying is primarily based on subjective measurements.

**OBJECTIVE:**

This cross-sectional field study with technical measurements aimed to quantify physical workload in terms of muscular activity and degree of forward bending during bricklaying at different working heights among masons, i.e., knee, hip, shoulder, and above shoulder height.

**METHODS:**

Twelve male (36.1±16.1 years) experienced masons participated in a cross-sectional field study with technical measurements. Surface electromyography from erector spinae longissimus and upper trapezius muscles and an inertial measurement unit-sensor placed on the upper back were used to assess the physical workload (level of muscle activation and degree of forward bending) different bricklaying heights. Manual video analysis was used to determine duration of work tasks, frequency, type, and working height. The working heights were categorized as ‘knee’, ‘hip’, ‘shoulder’, and ‘above shoulder’. The 95 percentiles of the normalized Root Mean Square (RMSn) values were extracted assess from erector spinae and trapezius recordings to assess strenuous level muscle of muscle activation.

**RESULTS:**

The RMSn of dominant erector spinae muscle increased from hip- to shoulder height (from 26.6 to 29.6, *P* < 0.0001), but not from hip to above shoulder height and decreased from hip to knee height (from 26.6 to 18.9, *P* < 0.0001). For the dominant trapezius muscle, the RMSn increased from hip- to shoulder- and above shoulder height (from 13.9 to 19.7 and 24.0, respectively, *P* < 0.0001) but decreased from hip- to knee height (from 13.9 to 11.5, *P* < 0.0001). Compared to hip height (27.9°), an increased forward bending was detected during bricklaying at knee height (34.5°, *P* < 0.0001) and a decreased degree of forward bending at shoulder- and above shoulder height (17.6° and 12.5°, *P* < 0.0001, respectively).

**CONCLUSION:**

Based on technical measurements, bricklaying at hip height showed the best compromise between muscular load and degree of forward bending. This study contributes to the development of the work environment for masons and can help guide preventive initiatives to reduce physical workload.

## 1 Introduction

Work-related musculoskeletal disorders (WMSDs), including low back pain and neck/shoulder pain, are a major concern for both workers, workplaces, and society [1–6]. WMSDs can reduce work ability and increase the risk of sickness absence and disability pension [7, 8]. Construction workers, including masons, are at high risk of WMSDs [9–14], particularly in the low back and shoulders [5, 9]. With more than 20,000 masons in Denmark [15], WMSDs are a significant problem that is influenced by individual factors as well as psychosocial and physical factors in the work environment [16]. Among all the risk factors, physical workload is a significant contributor to the risk of developing WMSDs [17, 18]. Masonry is characterised by repetitive movements, awkward postures, and heavy lifting [19, 20]. These physical work demands increase physical exertion and the risk of WMSDs, along with individual and psychosocial work factors [21–24]. The scientific literature indicates that repetitive work involving upper extremities increases the risk of pain in the arm and hand, carpal tunnel syndrome and tennis elbow [4, 25–28]. Particularly, the repetitive movements involving high force have been demonstrated to elevate the risk of pain [29]. A recent Danish cohort study involving 20,000 employees with a 2-year follow-up period revealed that neck-shoulder pain is increased among groups of workers where repetitive arm work is the predominant form of exposure [21].

A comprehensive study of the general working population in Denmark showed that painters, bricklayers, and plumbers report the highest overall physical work demands of all job groups [30]. In terms of specific postures, 82% of the masons reported working with twisted back more than 1/4 of their working time, and 60% working with arms at or above shoulder height [31]. However, these findings were based on self-reports in questionnaire surveys, which are prone to self-report bias [32, 33]. Therefore, to obtain a more detailed and accurate estimate of physical exposures in this type of work, technical measurements of exposure during real-world conditions are recommended [34]. The scientific literature regarding the physical work demands during masonry has shown that bricklaying is a physically demanding job [9, 35–41]. Although some previous studies have documented the physical demands of bricklaying, including muscle activity and posture, reported at various working heights [36, 38, 39], these studies employed substantial and heavy blocks that are incongruent with the lighter bricks commonly utilized in shell masonry.

### 1.1 Potential ergonomics risk assessments in construction

Valid and reliable measurement methods are essential for research of physical workload in the construction industry. Various technical measurement approaches exist, e.g. kinematics measured with inertial measurement units (IMUs), surface electromyography (sEMG), video recordings, and pressure-measurement insoles [42]. Kinematics involves studying body postures, often assessed using cost-effective IMUs [43]. *Inertial measurement units* have been used to e.g. detect body positions among construction workers [10, 44–46]. In contrast, sEMG offers insight into a worker’s level of muscular load, revealing the level of myoelectrical activity in skeletal muscles [47, 48]. sEMG has been used to evaluate the effects of lifting weights on the level of muscular load and the development of muscle fatigue during a simulated repetitive lifting task in a laboratory environment. and showed an increased level of muscular activation and muscle fatigue in e.g. the lumbar erector spinae muscle by increasing the lifting weights [49]. Furthermore, sEMG has been used to detect the level of muscle activity in field studies in among construction workers e.g. [44, 45, 48], to measure fatigue development e.g. [50] and to classify activity type e.g. [45, 51].

IMU-sensors have been used to detect body posture in the construction industry e.g. [45, 52] and onset/offset of activity e.g. [53, 54]. Furthermore, IMUs have been used for developing a real-time motion warning equipment for WMSDs prevention [55]. IMUs and sEMG have also been used in a combination to obtain a more thorough biomechanical evaluation at work e.g. [45, 52]. Besides sEMG and IMUs, wearable insole systems have been used to detect awkward working postures in construction workers [49, 56, 57].

This study aimed to quantify physical workload in terms of level of muscular activation and degree of forward bending of the trunk during work at different working heights among masons, i.e., knee, hip, shoulder, and above shoulder height. All measurements were performed at the respective work sites during their usual daily work. The study uses previously unpublished data collected in relation to a previously published cluster randomised controlled trial across workplaces in Denmark [45, 58]. Consequently, field measurements of physical workload among masons performing bricklaying were collected while the working height was systematically controlled and monitored by video recording. Simultaneous recording of the level of muscular activationof shoulder and back muscles as well as degree of forward bending were obtained.

## 2 Methods

### 2.1 Study design

This cross-sectional study with objective field measurements employs data from a two-armed, parallel group, single-blinded, cluster randomized controlled trial with allocation concealment performed at construction sites across Denmark from May 2016 to June 2017 [45, 58]. The present study re-analyses these data including only masons doing bricklaying.

### 2.2 Ethical considerations

According to the Helsinki declaration, participants received written and oral information about the purpose and content of the study before signing the informed consent form. The study was approved by the local ethical committee of Frederiksberg and Copenhagen (H-3-2010-062) and registered by the Danish Data Protection Agency (Datatilsynet; journal number 2015-57-0074). All data were processed and analysed anonymously.

### 2.3 Participants

Of the 80 full working days (∼7 h) of construction work in total, six working days were excluded due to poor data quality. During the remaining 74 working days, 12 experienced masons with bricklaying as their profession performed bricklaying and were thus included in the data analysis. In addition to the technical measurements, described below, the participants answered questionnaires on the days of the technical measurements.

### 2.4 Data collection

All measurements were performed at the respective work sites during their usual daily work. The technical measurement protocol in this study was based on previous studies [45, 58] and consisted of recordings of working situations using level of muscular activation from the erector spinae and upper trapezius muscles, kinematic information of body segments using IMU, and by a body worn video camera.

#### 2.4.1 Surface electromyography (sEMG)

**Fig. 1:**
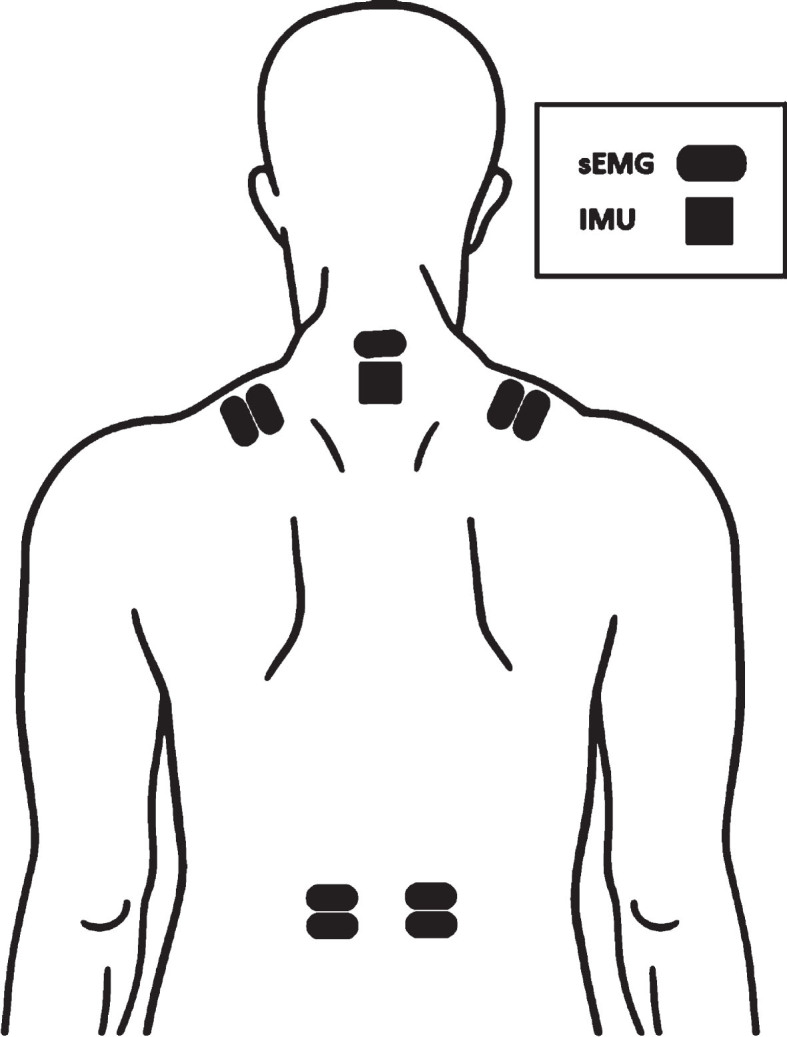
Placement of the surface electromyography (sEMG) electrodes over the dominant and non-dominant upper trapezius and erector spinae muscle and inertial measurement unit (IMU)-sensor. Of note the electrodes above the IMU served as reference electrode.

Surface electromyography electrodes (Blue Sensor N-00-S/25, Ambu A/S, Ballerup, Denmark) were placed bilaterally with a 2 cm inter-electrode distance over the m. erector spinae longissimus and the upper trapezius muscles (Fig. 1) according to the Surface Electromyography for the Non-Invasive Assessment of Muscles (SENIAM) recommendations [59]. The level of muscular activation of the erector spinae longissimus and upper trapezius muscles were recorded, as these body regions (lower back and neck/shoulders) are commonly prone to WMSD [60]. For the upper trapezius muscles, the electrodes were positioned at the approx. 20% lateral to the midpoint along the line connecting the acromion to vertebral spine C7. Regarding the erector spinae muscles, the electrodes were placed to the side of the L1 spinous process, at a distance approximately equivalent to the width of two fingers (about 2.5 cm) [45, 61]. The reference electrode was placed over the C7 vertebra. The skin of the participants was prepared to reduce skin impedance before placing the sEMG electrodes. To achieve this, the skin was shaved, cleaned using scrubbing gel (Acqua gel, Meditec, Parma, Italy), and then sanitized with surgical alcohol. The sEMG was sampled and digital transformed using a 24-bit portable data-logger (Nexus10, Mind Media, Herten, Netherlands) and sampled at 1024 Hz. The sEMG signals were digitally filtered using a bandpass (10 –400 Hz) 4^th^ order Butterworth filter and subsequently smoothed after computing root mean square (RMS) values over 500 ms epochs [62]. The sEMG signals were visually inspected for potential artifacts.

#### 2.4.2 Maximal voluntary isometric contractions (MVIC)

Each participant performed three maximal voluntary isometric contractions (MVIC) with one to two minutes of rest between trials to prevent fatigue. A custom-built dynamometer with a strain gauge load cell was used to measure MVIC (KIS-2, 2 KN, Vishay Transducers Systems, Malvern, PA, USA). For the low back muscles, a strap was placed around the upper back of the participants at the level of insertion of the deltoid muscle and connected to a strain-gauge dynamometer [63]. For the neck-shoulder muscles, the straps were placed around the participant’s wrists to a custom-built wooden plate with two strain gauge dynamometers. Participants were instructed to exert maximal force by building up force over a few seconds and exert maximally for another few seconds, while the test leader provided verbal encouragement. The maximum level of muscular activation of the erector spinae and trapezius muscle were extracted by computing the RMS valued over 500 ms epochs with 20% overlap between successive epochs. The maximum values were then used to normalize the level of muscular activation (% MVIC).

#### 2.4.3 Posture

Portable IMUs (ActiGraph GTX9-Link, ActiGraph, Pensacola, USA) were used to measure work posture. In this study, IMUs were used to detect the degree of forward bending. One IMU was placed on the upper back at T1-T2 level [61, 64]. The IMU were calibrated by having the subject standing with arms held neutrally besides the body for 15 seconds (Neutral pose) [65].

#### 2.4.4 Video recordings

Participants wore a wearable video camera to allow video recordings of their work tasks (Reveal Media, RS2-X2 L, Hampton Wick, Surrey, UK) synchronized to the sEMG and IMU measurements, which enabled to link the sEMG activity and IMU measurements with the working tasks performed during the workday. Therefore, the video recordings were only used during the technical analysis to detect the type, onset and offset of the identified working tasks

### 2.5 Technical analysis

All work tasks that included bricklaying were identified and time-stamped during the full working days for all masons using a custom-made MATLAB-programme by manually looking through all the video recordings. The start and stop for each work task were manually marked, and the frequency of the task, type of task, working height were identified. Working height was assessed as ‘floor height’, ‘floor to knee height’, ‘knee height’, ‘knee to hip height’, ‘hip height’, ‘hip to shoulder height’, ‘shoulder height’, and ‘above shoulder height’. For subsequent analyses, the brick-laying heights were divided into ’knee height’, ’hip height’, ’shoulder height’, and ’above shoulder height’. After identifying all work tasks, the trials were double-checked to secure correct identification and edit the potential incorrect identifications.

### 2.6 Data analysis

Forward bending of the back and muscle activity of the bricklaying tasks were analysed using MATLAB (version 2018a, The MathWorks). For each identified task the accelerometer data was low-pass filtered (6^th^ Butterworth) with a cut-off frequency of 5 Hz and the extent of forward bending/inclination of the back relative to neutral position was calculated [66]. The sEMG signals were visually inspected for potential artifacts, and any data with poor signal quality was subsequently removed from the dataset. The highest levels of muscular activation in the erector spinae and trapezius muscles were determined by calculating the RMS over a moving 500 ms interval. These maximum values were subsequently utilized to normalize the muscular activation levels (% MVIC). Erector spinae and trapezius muscle activity of each identified task was assessed by calculating the 95th percentiles of the normalized RMS values, which represent the data points below which 95% of the normalized RMS values fell in the entire dataset.

### 2.7 Statistics

A linear mixed model with repeated measures (SAS, Proc Mixed) [67] was used to determine the influence of lifting height on muscular workload. Normalized RMS (erector spinae and trapezius) and posture (degrees forward bending) were dependent variables. Brick laying height (knee, hip, shoulder, and above shoulder height) was used as the categorical independent variable. Individual was included as a random factor. Results are reported as least square means and 95% CI. When a significant main effect occurred, *P*-values were given for each respective work tasks in relation to brick laying at hip height (i.e., most ‘neutral’ working position) in pairwise comparisons. An alpha level of *P* < 0.05 was used as a statistically significant difference.

#### 2.7.1 Statistical power

Previous laboratory studies using EMG measurements demonstrated clear differences in muscular work load between different types of physical loads with 15–20 participants [68, 69]. In this project, field measurements were made during the working day, where there was expected a higher between-task variation [46]. Conversely, because the individual mason repeated every bricklaying work task several times during the working day, a more consistent estimate of exposure within-tasks was expected (i.e., more reliable measurement for each individual). Based on previous daily measurements with sEMG in workers with manual lifting work [46], it was estimated that at least 10 workers performing the respective work tasks were needed to have sufficient statistical power to show a difference in physical work load between different work tasks.

## 3 Results

Table 1 shows the characteristics of the participants. Twelve male masons with a mean age of 36 (±16.1) years participated in the study. For descriptive purposes, data on pain, physical tiredness, perceived exertion, number of steps, and heart rate are presented in Table 1.

**Table 1:** Characteristics of the participating masons

Mean	Std Dev
Number of participants	12 (all males)
Age (years)	36.1	16.1
Height (cm)	178.1	5.0
Body mass (kg)	78.2	12.7
BMI	24.6	3.5
Systolic blood pressure (mmHg)	136.1	13.7
Diastolic blood pressure (mmHg)	83.3	8.2
Morning pain in the back (NRS)	0.5	1.0
Afternoon pain in the back (NRS)	1.4	1.9
Morning pain in the shoulders (NRS)	0.7	2.0
Afternoon pain in the shoulders (NRS)	1.3	2.3
Morning physical tiredness in general (NRS)	1.1	1.3
Afternoon physical tiredness in general (NRS)	2.6	1.5
Perceived loading during working day (Borg)	3.8	1.9
Number of steps during working day	4859.7	1232.0
Mean heart rate during working day (bpm)	92.0	8.6
Maximum muscle strength in the back (N)	755.6	159.6
Maximum muscle strength in the shoulders (N)	137.4	34.2

Std Dev = Standard Deviation, mmHg = millimetre of mercury, NRS = Numeric Rating Scale, bpm = beats per minute, *N* = Newton.

### 3.1 Surface electromyography data

Table 2 presents 95^th^ percentiles of normalized RMS sEMG in the erector spinae and trapezius muscles of the dominant and non-dominant sides during masonry at knee, hip, shoulder, and above shoulder height and forward bending angle. The muscular activity for the dominant erector spinae muscle increased significantly from hip height to shoulder height (from 26.6% (15.6–7.7%) to 29.6% (18.6–40.6%), *P* < 0.0001), but not from hip height to above shoulder height. From hip height to knee height the muscular activity decreased (from 26.6% (15.6–7.7%) to 18.9% (7.9–29.9%), *P* < .0001). For the non-dominant erector spinae the muscular activity increased from hip height to shoulder height (from 24.6% (16.4–32.7%) to 30.8% (22.6–38.9%) (*P* < .0001), but not from hip height to knee- or above shoulder height.

**Table 2:** 95^th^ percentiles of the normalized root mean square values of the surface electromyographic recordings from the erector spinae and trapezius muscles of the dominant and non-dominant sides during masonry at knee, hip, shoulder, and above shoulder height and forward bending angle

Erector spinae				Trapezius			
Height	Dominant	*P*	Non dominant	*P*	Dominant	*P*	Non dominant	*P*	Forward bending	*P*
(upper back)
Knee height	18.9 (7.9–29.9)	< .0001	24.2 (16.0–32.4)	0.77	11.5 (7.4–15.5)	< .0001	12.4 (7.7–17.2)	0.0003	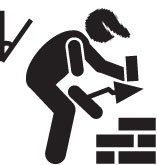	< .0001
									34.5° (40.3–28.3)
Hip height	26.6 (15.6–7.7)	Ref	24.6 (16.4–32.7)	Ref	13.9 (9.8–17.9)	Ref	15.9 (11.1–20.6)	Ref	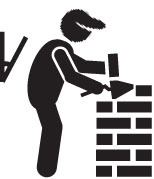	Ref
									27.9° (33.6–33.1)
Shoulder height	29.6 (18.6–40.6)	0.0002	30.8 (22.6–38.9)	< .0001	19.7 (15.6–23.8)	< .0001	25.0 (20.3–29.8)	< .0001	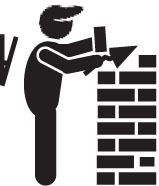	< .0001
									17.6° (23.3–11.9)
Above shoulder height	26.4 (15.3–37.4)	0.75	25.4 (17.2–33.5)	0.49	24.0 (19.9–28.0)	< .0001	31.4 (26.6–36.1)	< .0001	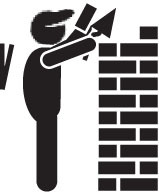	< .0001
									12.5° (18.2–6.7)

^*^P-values reflect the difference from hip height (reference/most neutral position).

For the dominant trapezius muscle, the muscular activity increased significantly from hip height to shoulder height (from 13.9% (9.8–17.9%) to 19.7% (15.6–23.8%) (*P* < .0001) and from hip height to above shoulder (13.9% (9.8–17.9%) to 24.0% (19.9–28.0%) (*P* < .0001) height but decreased significantly from hip height to knee height (from 13.9% (9.8–17.9%) to 11.5% (7.4–15.5%) (*P* < .0001). The same pattern was seen in the non-dominant side.

### 3.2 Inertial measurement units data

Furthermore table 2 shows an increased forward bending compared to hip height (27.9° (33.6–33.1)) during bricklaying at knee height 34.5° (40.3–28.3°) (*P* < .0001) and an decreased degree of forward bending at shoulder height 17.6° (23.3–11.9°) (*P* < .0001) and above shoulder height 12.5° (18.2–6.7°) (*P* < .0001).

## 4 Discussion

This study used wearable electromyography and inertial movement sensors during field measurements to assess the impact of degree of forward bending on physical workload of the low-back and neck-shoulder muscles among masons performing bricklaying work. The main findings were that bricklaying at and above shoulder height in general showed the highest level of muscular activation, while the back was least bent during these work tasks.

### 4.1 Construction work and biomechanical load

Bricklaying at knee height showed the highest degree of forward bending, while bricklaying at hip height showed the best compromise between muscular load and degree of forward bending, at least according to the results from the present study. These findings have the potential to inform preventive measures aimed at reducing physical workload during bricklaying. The present study showed an even distribution on the physical workload between the dominant and non-dominant side (Table 2) as well as an increased physical workload on the neck/shoulder muscles when the bricklayers performed their work above shoulder height. Previous research manifested similar findings during a simulated drilling task [70] and among a convenience sample of supermarket workers [71, 72]. Occupational lifting with arms above shoulder height resulted in higher muscular workload based on sEMG measurements and higher compression forces in the shoulder joints based on a musculoskeletal modelling approach [71, 72]. The above mentioned findings elaborate on previous studies showing that elevated arm work increases the biomechanical load of the shoulder muscles and restricts the blood supply to shoulder and arm tissues [73]. Working with arms above shoulder level increases the risk of excessive fatigue, musculoskeletal pain, and long-term sickness absence [22, 44, 74].

### 4.2 Technical measurements in construction work

The present study uses technical field measurements that have been used in previous research in the construction industry [54, 75, 76] to assess physical workload during bricklaying. Previously, researchers have studied physical workload in terms of; workload, posture, heart rate, and oxygen uptake during bricklaying [35, 36, 40, 41]. Van der Molen and co-workers studied the physical work demands and experienced local discomfort of the low back and shoulders in a controlled field study where they investigated the effects of adjusting working height of bricks and mortar. They demonstrated a reduction in the frequency and duration of trunk flexion > 60° when introducing a scaffolding console that increased the height of the bricks and mortar with 31 cm [40]. Vink & Koningsveld found that the heightened the placement of bricks and mortar appeared to lower the energy consumption, especially when bricks were placed in higher rows in the wall [41]. Because these studies did not include technical measurements of the physical load in terms of posture and muscle activity, the present study makes a genuine contribution to the field by providing objective knowledge about the physical workload during bricklaying. Anton and co-workers showed an increasing muscle activity in the shoulder muscles when bricklaying heights were increased [35, 36]. Even though, these studies used concrete blocks, the result are in line with the present study. Based on the collective results from the present and previous studies, masons should endeavour not to lay bricks at low heights and increase the height of the placement of bricks and mortar to decrease the load on the low back. The present study provides evidence of a “safety margin” [77] for bricklayer workers that can contribute to decrease their physical exposure and improve their working conditions.

In Denmark, the Work Environment Authority recommends bricklayers to avoid bricklaying above shoulder level [78], although this regulation is not based on technical measurements. Importantly, the present study using technical field measurements support previous results based on self-reports [9]. Thus, the present study validates existing recommendations. Furthermore, Gupta and co-workers found an exposure-response association between working with elevated arms above shoulder level (measured with accelerometers) and long-term sickness absence based on high-quality national registers in a prospective cohort study [33].

### 4.3 Study implications and contributions

When evaluating the practical implications of the study results, both muscular load and posture should be taken into account. Masons should avoid bricklaying above shoulder height when possible. However, situations may occur where bricklaying above shoulder level is unavoidable, e.g., during floor separations. Likewise, working at knee height resulted in a more flexed trunk posture, which is a known physical risk factors for the development of WMSDs in the low back region [79, 80]. In these cases, workplaces should prioritize the use and development of technical devices that can reduce the physical workload to minimize the risk for developing future WMSDs. Rotating between workers may also be an option to avoid that the same workers are always exposed to the most difficult working situations [81].

### 4.4 Strengths and limitations

The strength of the present study is that it comprised data from technical measurements obtained from masons performing their specific work in field conditions [82] resulting in a high external validity. Due to the high number of repeated measurements in each worker –i.e., laying several bricks during the workday –muscular workloads and working postures were measured with high precision. The repeated measures also increased the statistical power enabling to detect relatively small differences in normalized sEMG between conditions. However, the study also has some limitations. The study only included relatively young males, and the results may, therefore, not be generalized to female or older workers due to the fact that there are differences in anthropometry, biology, and lifting strategies between gender [83, 84]. However, there were no gender-specific inclusion- or exclusion criteria and the recruited participants reflect the fact there are very few female bricklayers in Denmark. Another limitation is that only level of muscular activation data from the lower-back and neck/shoulder muscles was collected even though masons primarily report musculoskeletal complaints in these two regions. Thus, the level of muscular activation did not enable to assess changes in muscle coordination of the lower-back and neck/shoulder region A third limitation is that the present study did not explore the level of muscular activation patterns over time during the working day. While this would require new analyses, the present dataset holds the potential to explore such patterns. Wearable insoles is another possibility to detect load lifted or detecting the working situations in construction [49, 56, 57], and including these measurements could potentially have added information about the load of the brick and mortar.

## 5 Conclusion

This study cross-sectional field study quantified physical workload in terms of muscular activity and degree of forward bending during masonry work at different working heights, i.e., knee, hip, shoulder, and above shoulder height. Technical measurements in terms of sEMG, IMUs, and video recording were used to document the physical workload at and above shoulder height showing that the highest level of muscular activation in the non-dominant trapezius 25.0% (20.3–29.8%) and 31.4% (26.6–36.1%), respectively), while this working position constituted the most upright standing position (i.e., least forward bending). Bricklaying at knee height showed the highest degree of forward bending (34.5° (40.3–28.3°) compared to hip height (27.9° (33.6–33.1°). Bricklaying at hip height showed the best compromise between level of muscular activation and degree of forward bending. These results can help guide preventive initiatives to reduce physical workload during bricklaying.

## Ethical approval

The study was approved by the local ethical committee of Frederiksberg and Copenhagen (H-3-2010-062).

## Informed consent

According to the Helsinki declaration, participants received written and oral information about the purpose and content of the study before signing the informed consent form.
